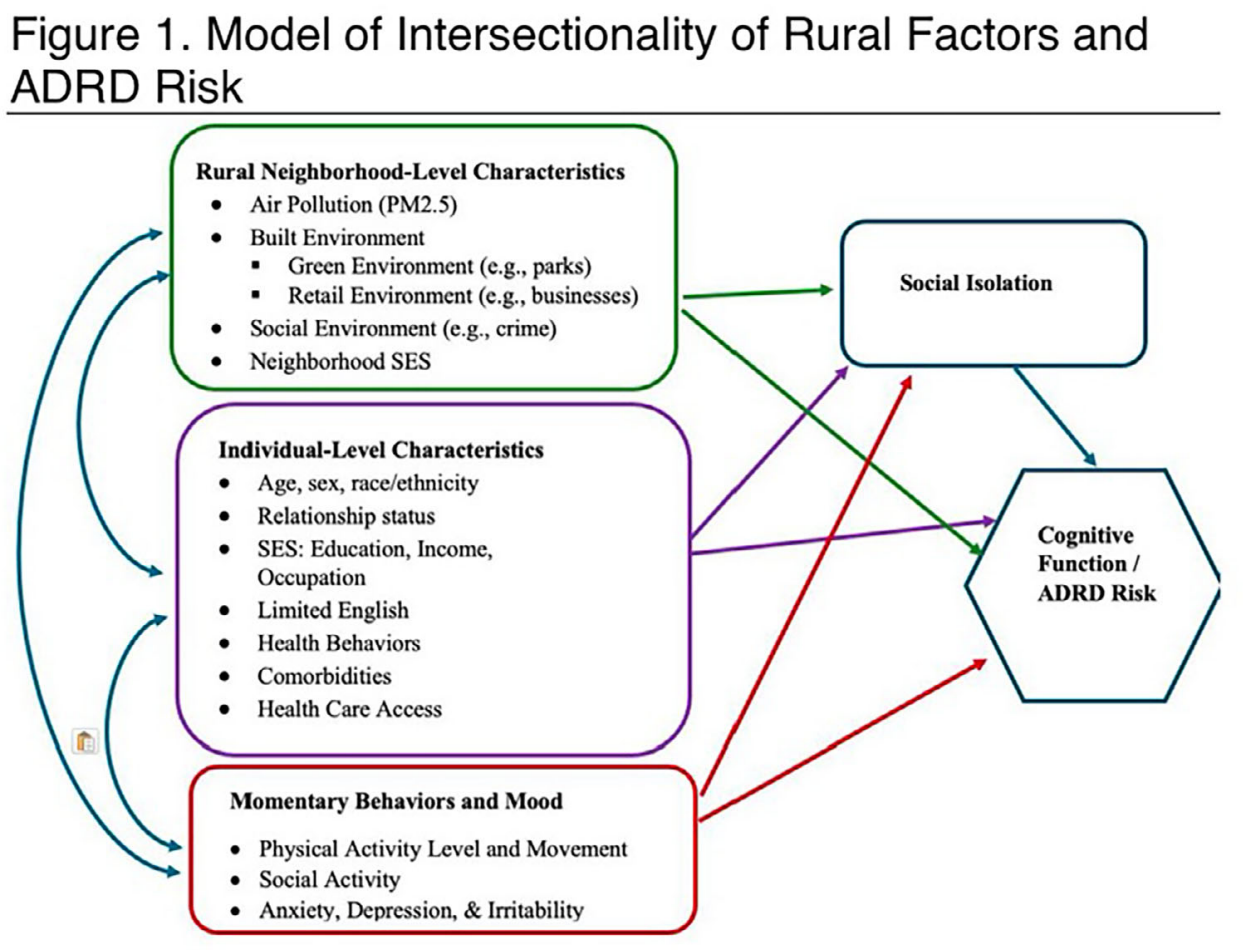# Relationships among cognitive function, ADRD risk and sociodemographic characteristics in a diverse rural setting in south‐central Florida

**DOI:** 10.1002/alz70860_101721

**Published:** 2025-12-23

**Authors:** Christine L. Williams, Lisa Ann Kirk Wiese, Janet Holt, Lilah M Besser

**Affiliations:** ^1^ Florida Atlantic University, C.E. Lynn College of Nursing, Boca Raton, FL, USA; ^2^ C.E. Lynn College of Nursing, Florida Atlantic University, Boca Raton, FL, USA; ^3^ University of Miami Miller School of Medicine, Boca Raton, FL, USA

## Abstract

**Background:**

Previous research has shown that cognitive function often declines in mid to late life however, rural and ethnoracial minority groups are more likely to experience conditions that contribute risk of ADRD. Our participants from five rural communities surrounding Lake Okeechobee, FL experience a high burden of chronic illness and low healthcare access. This community‐engaged, resident‐led study was made possible through relationships over the past nine years involving service and research projects.

**Method:**

Over 12 months, participants were screened with the mini‐Moca to determine eligibility for the study (adjusted score of 12 or higher). Our primary outcome, cognitive function, was measured by the MoCA and subjective memory decline. ADRD risk was measured by the mCAIDE. Sociodemographic characteristics included age, sex, education, ethnicity, and years lived in study settings. Neighborhood vulnerability was measured with the Area Deprivation index (ADI).

**Result:**

Participants (*n* = 399) have completed the first follow‐up assessments, including MoCA, mCAIDE, memory self‐report, demographic information. Most identified as female (287, 72%). Ethnic group membership included Black (311, 78%), White (74, 19%), Hispanic/Latino (61, 15%), Afro‐Caribbean (12, 3%) and 14 (4%) reported other race(s). Median age was 64 years, with 12 years of education and residence in study communities, 50 years. Notably, 203 (51%) had MoCA scores of 26+, while 150 (38%) reported memory decline. None of the covariates predicted subjective memory decline, *p* < .05. Of the 374 participants with relevant data, education and Hispanic/Latino ethnicity were significant and positive predictors of MoCA scores (*p* < .001), while age, gender, years in study communities, and the area deprivation index were not significant predictors (*p* > .05). Of the 359 participants with relevant data, age (*p* < .001) and education (*p* = .007) were significant and positive predictors of mCAIDE scores, while gender, Hispanic/Latino ethnicity, years lived rural, and the ADI were not significant (*p* > .05).

**Conclusion:**

Approximately half the participants are at‐risk for ADRD based on MoCA scores. Although participants with more education and/or Hispanic/Latino ethnicity experienced less ADRD risk, there is a need to identify environmental factors that cognitive decline among rural residents.